# Ferroelectric 2D ice under graphene confinement

**DOI:** 10.1038/s41467-021-26589-x

**Published:** 2021-11-01

**Authors:** Hao-Ting Chin, Jiri Klimes, I-Fan Hu, Ding-Rui Chen, Hai-Thai Nguyen, Ting-Wei Chen, Shao-Wei Ma, Mario Hofmann, Chi-Te Liang, Ya-Ping Hsieh

**Affiliations:** 1 Institute for Atomic and Molecular Science, Academia Sinica, Taipei, 10617 Taiwan; 2 International Graduate Program of Molecular Science and Technology, National Taiwan University, Taipei, 10617 Taiwan; 3 Molecular Science and Technology Program, Taiwan International Graduate Program, Academia Sinica, Taipei, 10617 Taiwan; 4 Department of Chemical Physics and Optics, Faculty of Mathematics and Physics, Charles University, Prague, 121 16 Czech Republic; 5 Department of Physics, National Taiwan University, Taipei, 10617 Taiwan; 6 Department of Materials Science and Engineering, National Cheng Kung University, Tainan, 70101 Taiwan; 7 Graduate Institute of Opto-Mechatronics, National Chung Cheng University, Chiayi, 62102 Taiwan

**Keywords:** Graphene, Ferroelectrics and multiferroics, Surfaces, interfaces and thin films, Electronic and spintronic devices

## Abstract

We here report on the direct observation of ferroelectric properties of water ice in its 2D phase. Upon nanoelectromechanical confinement between two graphene layers, water forms a 2D ice phase at room temperature that exhibits a strong and permanent dipole which depends on the previously applied field, representing clear evidence for ferroelectric ordering. Characterization of this permanent polarization with respect to varying water partial pressure and temperature reveals the importance of forming a monolayer of 2D ice for ferroelectric ordering which agrees with ab-initio and molecular dynamics simulations conducted. The observed robust ferroelectric properties of 2D ice enable novel nanoelectromechanical devices that exhibit memristive properties. A unique bipolar mechanical switching behavior is observed where previous charging history controls the transition voltage between low-resistance and high-resistance state. This advance enables the realization of rugged, non-volatile, mechanical memory exhibiting switching ratios of 10^6^, 4 bit storage capabilities and no degradation after 10,000 switching cycles.

## Abstract (web summary)

Ferroelectric ordering of water has been at the heart of intense debates due to its importance in enhancing our understanding of the condensed matter. Here, the authors observe ferroelectric properties of water ice in a two dimensional phase under confinement between two graphene layers.

## Introduction

Ferroelectric ordering of water has been at the heart of intense debates for nearly a century^1–3^ due to its importance in enhancing our understanding of condensed matter. Despite significant research efforts, no clear evidence of water ice with the necessary proton ordering has been reported leading to the coining of the term “UFI” (underidentified ferroelectric ices) in the literature^4^.

Ferroelectric ordering in ice is challenging to observe experimentally, due to the a large number of energetically equivalent arrangements of water molecules and the small energy differences between disordered and ordered ice forms. The presence of a suitable surface could change the energetics and kinetics of water ordering as evidenced by fast water transport in nanotubes^5^, exotic water morphologies in atomic pores^6^, and new thermodynamic phases on graphene surfaces^7–9^. Theoretical predictions suggest that the confined conditions between two graphene layers could generate atomic layers of ice with a preferential dipole orientation that results in ferroelectric ordering^10,11^.

Experimental characterization of of such 2D ice monolayers in contact with graphene is hindered by issues in their production and characterization^12,13^ and UFIs remain at large.

We here demonstrate direct evidence for the formation of ferroelectric ice when water is actively confined to a monolayer between two layers of graphene. Nanoelectromechanical actuation is employed to provide intimate and controllable contact between two graphene layers and to generate a polarization across their interface. A field-dependent dipole ordering was observed by three separate experimental techniques which is retained even after the field is removed. The ferroelectric response of the ice layer vanishes upon the formation of ice multilayers, indicating the unique properties of 2D ice.

The robust and room-temperature stable ferroelectric response of ice at the graphene interface not only represents a new phase of water but can be applied to impart nanoelectromechanical devices with unexpected properties. This advance is demonstrated by a mechanical switch that exhibits an unexpected bipolar switching behavior where a positive voltage toggles a low resistance state and a negative voltage is necessary to reset the switch to a high resistance state and enables the first realization of two-terminal mechanical memory. Moreover, the reset voltage at which the transition between the resistance states occurs is found to depend on the history of the previously applied voltage, giving rise to a unique memristive effect, which was employed to realize a 4 bit multi-level mechanical data storage device.

## Results

While all surfaces are covered by a water film under ambient conditions, forming a monolayer of water is a complex process that is difficult to control outside of ultrahigh vacuum conditions. Self-organization effects at the graphene interface can produce islands of monolayer water but have no control over their position and extent^7,14–16^. Moreover, previous work demonstrated that the slow lateral diffusion at the 2D materials interface leads to thick water clusters instead of continuous thin films^17^.

**Fig. 1:**
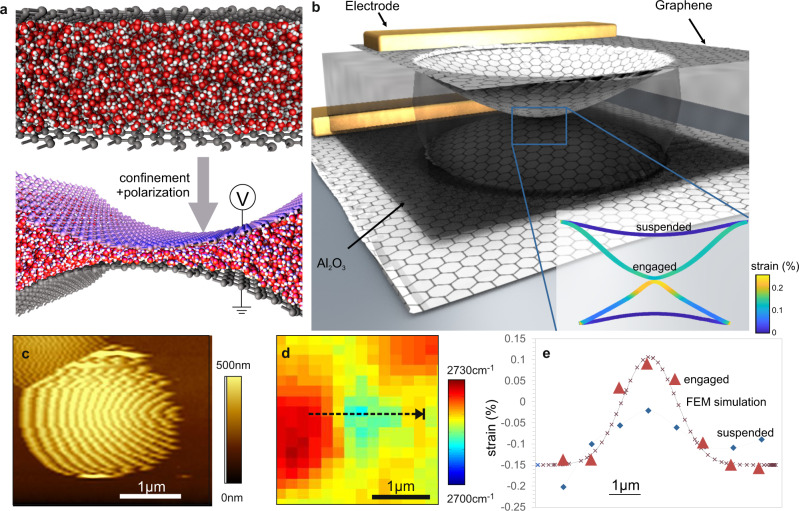
Nanoelectromechanical actuation scheme. (**a**) concept of nanoelectromechanical confinement of water between graphene, (**b**) schematic of employed device structure, (Inset) finite-element simulation of deformation and strain distribution upon contact (**c**) tapping mode AFM image of suspended top layer over drum structure, (**d**) map of Raman 2D band position, (**e**) strain distribution extracted from Raman scaling analysis corresponding to the cross-section in (**d**).

We pursue a more robust approach to forming ultra-thin water films based on a nanoelectromechanical (NEMS) actuation approach where two separated graphene layers are brought into contact by electrostatic attraction. This dynamical adjustment of the graphene layer separation permits the variation of the inter-layer pressure to displace excess water from the contact point between the two graphene layers (Fig. 1(a)).

A lithography-free fabrication process was devised to produce micrometer-scale drum structures (Fig. 1(b)) while minimizing contamination of the graphene interfaces as detailed in the supplementary materials. Atomic force microscopy demonstrates the formation of suspended graphene as evidenced by oscillations in the image signifying superposition of imaging and resonator vibration frequency^18^ (Fig. 1(c)).

The condition of the graphene electrode can be investigated by spatially resolved Raman spectroscopy (Fig. 1(d)). A redshift of the Raman 2D-band position indicates that the suspended portion of the graphene is strained compared to the supporting part, due to sagging, wall adhesion, and capillary forces^19,20^. The strain can be quantified by Raman scaling analysis^21^ and we extract the evolution of strain on the suspended graphene (Fig. 1(e)).

Next, a voltage is applied between the bottom and top graphene layer and subsequently removed. Raman spectroscopy is then carried out again at the same location and we observe an increased strain in the suspended membrane compared to initial conditions (Fig. 1(e)). The magnitude and distribution of strain is in good agreement with finite element simulations for contacted graphene membranes in the described geometry (inset Fig. 1(b), more details in the Supplementary Information).

**Fig. 2:**
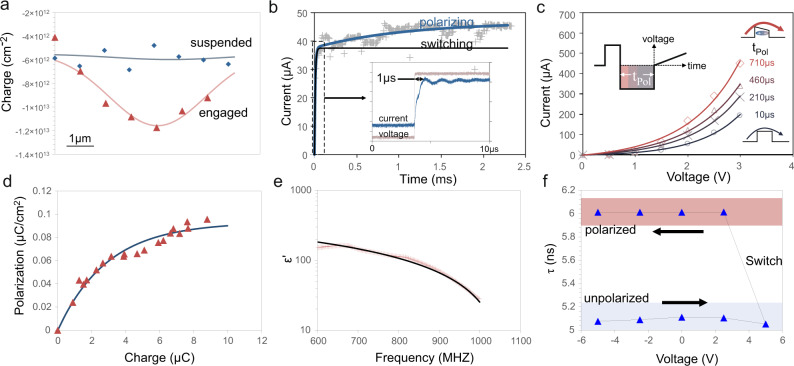
Observation of ferroelectric polarization. **a** Spatial charge distribution across drum from Raman scaling analysis of drum in Fig. 1(d), (**b**) time-resolved current measurement with fits to switching ($\tau =1\,\mu {{{\rm{s}}}}$) and polarization ($\tau =1\,{{{\rm{ms}}}}$) processes, (inset) high speed measurement of switching transition, (**c**) current-voltage characteristics obtained by PUVD technique at different polarization conditions (inset) schematic of PUVD scheme, (**d**) time evolution of polarization with charging, (**e**) high-frequency measurement of dielectric constant with fit to Debye relaxation model, (**f**) plot of extracted relaxation time constant $\tau$ during switching.

In addition to moving the graphene electrodes in contact, the one-time application of voltage significantly modifies the charge distribution along the graphene membrane even after the bias has been removed. Raman analysis reveals a doubling of the p-type doping in the center of the suspended area, compared to the pristine condition (Fig. 2(a)). This observation suggests the formation of a permanent and sizable dipole when graphene layers are brought into intimate contact and provides the first evidence of the ferroelectric nature of the structure.

To confirm the ferroelectric properties of the assembly we conduct electrical transport measurements which help identify permanent changes in its capacitance. As the capacitance change of an individual device is expected to be extremely small^22^, we investigate the response of large device arrays where many switches are connected in parallel through a common graphene conduction pathway (more information on device fabrication and measurements are provided in the supplementary material). When applying a variable, high-frequency excitation to this multi-drum array, a single and sharp resonance peak at 246 MHz is observed (Suppl. Fig. S5), which confirms that individual drums are not interacting with each other and validates our approach (more information about the RF resonator measurements are provided in the supplementary material).

Time-resolved measurements of the mechanical contracting process between the two graphene membranes show a sharp increase in current within $1\,\mu {{\rm{s}}}$ after an electrical force is applied (inset Fig. 2(b)). This transition indicates the formation of mechanical contact between the two graphene layers. The high switching speed compared to other double-cantilevered NEMS switches^23^ indicates the low mass and high strength of the graphene membrane.

After the mechanical switching process is complete, however, the current keeps increasing with a longer characteristic time constant of ~1 ms. To provide evidence for the formation of a ferroelectric dipole, we devise a modified “positive up, variable down” (PUVD) measurement technique. In conventional “positive up, negative down” PUND measurements, two sets of polling pulses are used to switch the dipole orientation and the concomitant charge flow is analyzed. However, this PUND approach is complicated by the presence of electrical (leakage) currents, changing contact geometries, and small capacitance values as expected for the employed NEMS device^24^. We, instead, investigate the changes in tunneling electroresistance at different polarization conditions (inset of Fig. 2(c))^25^. A non-linear IV curve confirms the presence of an injection barrier and for longer polarization times (t_Pol_) this injection barrier is found to be lowered which is in agreement with through the formation of an opposing polarization dipole in the  ferroelectric picture. The change in injection barrier height can be related to the ferroelectric polarization^22^ (as detailed in the supplementary material) and we observe an initially monotonic increase in polarization with applied charge and a subsequent saturation (Fig. 2(d)) that confirms the ferroelectric nature of the process.

A third evidence for ferroelectric ordering is obtained by high-frequency measurements. Variable-frequency excitation reveals a monotonically changing dielectric constant (Fig. 2(e)) which indicates a single relaxation mode that follows a simple Debye model^26^. The relaxation time constant was extracted at different switching conditions (see supplementary material). We find that there is a hysteresis in the time constant with applied voltage (Fig. 2(f)) that shows a sharp change from a that is stable once generated. A low to a high relaxation time constant agrees at a high switching voltage which agrees with the transition from amorphous water to ice^26^. This effect is indicative of the change in polarization after the graphene is brought into contact and thus confirms the presence of ferroelectric ordering that is stable once generated.

**Fig. 3:**
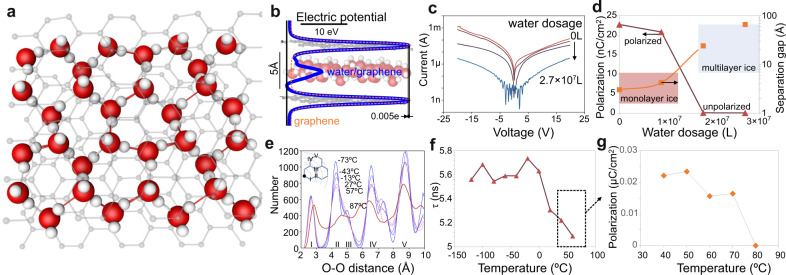
Origin of ferroelectric effect. (**a**) DFT simulation results of confined water relaxed structure displaying ice XI configuration, (**b**) electrostatic potential distribution (relative to origin) along the z-axis of pristine graphene and graphene/ice structure with overlaid 3D image of relaxed structure (**c**) current-voltage characteristics of the device under different amounts of water coverage, (**d**) tunneling distance between electrodes and polarization as a function of water coverage, (**e**) bond distance histogram of structures relaxed at different temperatures through molecular dynamics simulation, (**f**, **g**) Temperature dependence of (**f**) high-frequency relaxation time constant and (**g**) PUVD polarization.

Ab-initio simulations were conducted to establish the origin of the observed ferroelectric behavior. For this purpose, the stability of monolayer water confined between two graphene layers was studied (Fig. 3(a)). Due to the experimentally observed significant change transfer upon confinement, we consider the polarization of graphene through reorientation of water molecules under electric fields that are comparable to the experimental values in our DFT approach (more details in the supplementary material). We observe that ice XI, the most promising configuration of ferroelectric ice, is indeed stable on graphene and that the dipole-aligned phase, when confined to an ice monolayer, exhibits high stability. This observation supports our experimental results that ferroelectric behavior, once induced, can remain stable.

The preferential alignment of the water dipoles produces a difference in polarization between the top and bottom layer of graphene that is calculated to be approximately 0.0034 electrons/carbon atom (Fig. 3(b)). This polarization agrees with our extracted charge transfer from Raman measurements $\frac{\Delta n}{{c}_{{carbon}}}=0.0016\frac{{electrons}}{{carbon\; atom}}$ (Fig. 2(a)).

Furthermore, our simulations corroborate the change in carrier transport mechanism assumed for our PUVD analysis. With the increasing alignment of water dipoles, an asymmetric electrostatic potential develops between layers that lowers the injection barrier in agreement with experimental results (Fig. 3b).

To experimentally confirm the role of water in the observed ferroelectric response, we conduct transport measurements at variable water exposure. A device is initially baked in a vacuum to remove water and then water is introduced into the vacuum at a slow rate by a leak valve. We observe a significant change in carrier transport during this process (Fig. 3(c)). First, a decrease in overall current can be attributed to an increase in tunneling distance between the graphene contacts as the water layer thickness increases. Second, a change in differential conductance is observed that suggests a variation of carrier injection upon water exposure.

To quantify these observations, we apply the previously described PUVD analysis approach to each IV (more details are provided in the supplementary material). The tunneling distance is found to increase with water exposure in a range from 6 to 25 A which represents a range of separations between monolayer and multilayer ice XI (Fig. 3(d))^15,27^. We find that the polarization abruptly decreases as the water-induced layer separation reaches multilayer thickness (Fig. 3(d)). This result suggests that a bilayer of ice loses its ferroelectric response which agrees with our DFT simulations that demonstrate that the dipole-aligned phase exhibits a lower stability than the random-dipole phase by 60 meV per water molecule.

The remarkable stability of the ferroelectric ordering of a confined single ice layer is further evident when studying the effect of temperature. Molecular dynamics simulations demonstrate a loss of long-range order when temperatures are increased to 87 °C which indicates the conversion of crystalline ice into water (Fig. 3(e)).

This prediction is confirmed by two experimental techniques. First, high-frequency measurements reveal a linear decrease in relaxation time constant with a temperature above 0 °C (Fig. 3(f)), which supports the gradual transition from the ferroelectric ice phase to water akin to soft ferroelectric materials^28^. This loss of a dipole ordering is further supported by PUVD measurements (Fig. 3(g)) that demonstrate a complete suppression of the ice phase around 80 °C, which agrees with our simulation and previous reports^29^.

**Fig. 4:**
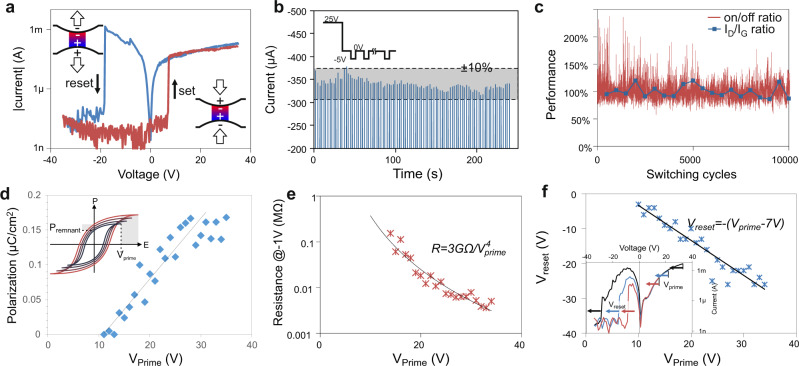
Nanoelectromechanical memristor. **a** current-voltage characteristics of a mechanical switch demonstrating a novel bipolar switching behavior, **b** change of electrical performance (on/off ratio) and defectiveness (Raman I_D_/I_G_ ratio^32^) during repeated cycling (**c**) current after single write cycle at 25 V and various delay and read cycles at 0 V and −5 V, respectively, (inset) representation of pulse sequence, (**d**) remnant polarization as a function of previously applied highest voltage (V_prime_), (inset) representation of polarization hysteresis, (**e**) evolution of device resistance depending on previously applied priming voltage, (**f**) change of reset transition voltage as a function of previously applied priming voltage.

In addition to answering fundamental questions, the observed ferroelectric nature of confined water provides a route towards unprecedented applications. The hysteretic property of the dipole alignment leads to a situation  under reverse bias polarity where the applied electric field is opposed by the previously formed ferroelectric dipole (inset of Fig. 4(a)). Consequently, a repulsive force is exerted between the two graphene layers that pushes them apart. Due to this condition, our ferroelectric NEMS device exhibits a bipolar-like switching behavior where the graphene layers contact at positive voltages and separation occurs at negative voltages (Fig. 4(a)). This behavior is fundamentally different from other electromechanical switches where the reset transition is always at lower positive voltages than the set transition and, consequently, power has to be permanently supplied to keep the electrodes in contact. Consequently, the ferroelectric NEMS device can be employed as a permanent and non-volatile memory as evidenced by the demonstrated long retention time of information at power-cycled conditions (Fig. 4(b)).

Mechanical memory could provide improved reliability^30^ and power efficiency^31^ compared to conventional memory. We demonstrate the ruggedness of the system by cycling the device for 10,000 times (Fig. 4(c)). We observe that neither the electrical performance nor the materials defectiveness changes significantly throughout the operation which is due to the high mechanical strength and chemically inert nature of the graphene.

One important challenge to the application of mechanical memory is their low integration density. However, the presented ferroelectric functionality has the potential to overcome this limitation. The dependence of a ferroelectric’s remnant polarization on the previously applied electric field represents a memristive system that could enable multibit operation. We demonstrate this behavior in confined ice by applying a variable polarizing voltage and extracting the polarization through our modified PUVD. We observe a clear proportionality between the previously maximum applied electrical potential (termed priming voltage V_prime_) and the remnant polarization (Fig. 4(d)) that serves as further confirmation of the ferroelectric origin of the observed phenomenon.

This tunability of the interfacial polarization by a previously applied priming voltage is employed to adjust the device’s contact resistance. We observe a proportionality of the resistance measured at small reading voltages to the priming voltage over a large range that can be described by a simple empirical formula (Fig. 4(e)). The device thus acts as a multilevel memory where the measured resistance can be used as an analog to the previously encoded voltage. Even without optimization of the device performance, we can reliably encode 16 different resistance levels suggesting the use of the device as a multi-level memory (more information is provided in the supplementary material). Consequently, a single switch can hold at least 4 bits of data and thus increase the information storage density for a given device density.

An alternative operation mechanism is a voltage-controlled operation. In addition to modifying the injection barrier, the remnant polarization determines the electrostatic repulsion needed to overcome stiction between the two layers, i.e. a larger electric field needs to be applied to cancel out the effects of high polarization. We observe a clear dependence between the initial polarizing field and the reset voltage (Fig. 4(f)) at which the membranes separate. The observed linearity of the two parameters over a large range indicates the negligible amount of non-remnant polarization and suggests the dominance of single-crystalline ferroelectric domains in the response.

The voltage-controlled operation represents a memristive behavior that is fundamentally different from previous mechanisms. Conventional memristors exhibit a relation between charge and device resistance, whereas our ferroelectric NEMS device exhibits a relationship between charge and transition voltage. This difference could simplify current circuits as our device is found to switch into the off-state at increasing voltages (inset of Fig. 4(f)), whereas conventional memristors become more conductive. This self-limiting current characteristic could be employed as an adjustable current limiting element in circuits replacing the pervasive 1T1R (1 transistor 1 resistor) schematics to simplify RRAM crossbar architectures.

In conclusion, we have demonstrated the ferroelectric nature of water that is confined between two graphene layers. Several experimental approaches confirm the formation of a sizable and stable dipole that originates from a single layer of ice. The dipole strength and temperature stability are found to quantitatively agree with simulation results for the 2D phase of ice XI.

The demonstrated combination of mechanical and ferroelectric effects in a nanoelectromechanical switch yield a unique device that exhibits a bipolar-type switching characteristic and represents a novel memristor type. These advances not only provide new insight into the structural properties of nano-confined water but provide a new approach to high-density memory devices and future electronics.

## Methods

Graphene was synthesized on copper foil using chemical vapor deposition following previous reports^33^. Briefly, the copper foil was electrochemically polished at 2 V for 30 minutes and then annealed at 1000 °C for 2 h at 10 Torr under a flow of 10sccm hydrogen in a 1” quartz tube heated by a clamshell furnace. Then, 2sccm of methane was introduced to initiate graphene growth for a duration of 4 hours. Finally, the sample was cooled down to room temperature under a flow of 10sccm hydrogen.

To produce the double-membrane structure, the first layer of graphene was transferred on silicon wafers with a 300 nm thermal oxide using a wet transfer technique. For this process, PMMA was spin-coated at 2000 rpm onto the graphene-coated copper foil. The sample was then immersed in Ammonium persulfate to remove the copper foil and cleaned by multiple immersions in water. The floating membrane was then scooped up onto the wafer and dried before immersion in acetone to remove the PMMA layer.

After the deposition of the dielectric a second layer of graphene was transferred using a similar wet-transfer technique. To avoid capillary effects that might force the two graphene layers in contact we do not immerse the sample in Acetone for PMMA removal. Instead, Acetone vapor was employed to remove the PMMA layer. The resulting graphene exhibited high quality as identified by Raman spectroscopy (Supplementary Fig. 2).

Silicon microspheres of 1 µm diameter were deposited from ethanol solution (1 mg/l) by casting 1 ml and subsequent heating to 100 °C. Then, aluminum oxide was deposited by electron beam evaporation onto the first layer of graphene (Supplementary Fig. 1(a)). An optimized thickness of 40 nm was found to allow contact between graphene layers while preventing permanent stiction. Finally, the microspheres were removed by brief sonication of the sample in isopropyl alcohol.

The resulting structure exhibits well-separated drums as confirmed by optical microscopy over large areas (Supplementary Fig. 1(c)).

## Supplementary information

Supplementary Information

## Data Availability

Relevant data supporting the key findings of this study are available within the article and the Supplementary Information file. All raw data generated during the current study are available from the corresponding authors upon reasonable request.
