# Supplementary material for: Ferroelectric 2D ice under graphene confinement
Source: Nat Commun. 2021 Nov 1;12:6291. doi: 10.1038/s41467-021-26589-x (PMC8560911; doi:10.1038/s41467-021-26589-x)
Supplement: Supplementary file 1 — Supplementary Information [file 41467_2021_26589_MOESM1_ESM.pdf]

Supplementary Material for

Ferroelectric 2D ice under graphene confinement

Authors: Hao-Ting Chin[1,2,3], Jiri Klimes[4], I-Fan Hu[1,5], Ding-Rui Chen[1,2,3], Hai-Thai Nguyen[5,6], Ting-Wei Chen[7], Shao-Wei Ma[7], Mario Hofmann[5], Chi-Te Liang[5] and Ya-Ping Hsieh[1]*

Affiliations:

[1] Institute for Atomic and Molecular Science, Academia Sinica, Taipei, 10617 Taiwan

[2] International Graduate Program of Molecular Science and Technology, National Taiwan University, Taipei, 10617, Taiwan

[3] Molecular Science and Technology Program, Taiwan International Graduate Program, Academia Sinica, 10617, Taiwan

[4] Faculty of Mathematics and Physics, Charles University, Prague, 121 16 Czech Republic

[5] Department of Physics, National Taiwan University, Taipei, 10617 Taiwan

[6] Department of Materials Science and Engineering, National Cheng Kung University, Tainan, 70101 Taiwan

[7] Graduate Institute of Opto-Mechatronics, Chung Cheng University, Chiayi, 62102 Taiwan

*Corresponding author. Email:yphsieh@gate.sinica.edu.tw

Details on finite element simulation

Simulation was conducted in a three-dimensional geometry that is based on microscope analysis (Figure 1, Supplementary figure S1). We simulate a structure that contains a 1µm opening in a 40nm dielectric separation layer. The walls of the opening were angled at 140° to account for shadowing effects during evaporation (Supplementary figure S3). Graphene membranes were added to both bottom and top of the separator and were represented by a 1nm thick layer with an elastic modulus of 0.96TPa following previous reports.[1]

The graphene's lateral movement was constrained at the edge of the opening. Increasing voltages were applied to both graphene layers until contact was observed.

Upon contact, the strain distribution was extracted from the cutting line through the center of the orifice and convoluted with a gaussian function of FWHM of 1um to provide correspondence with the Raman measurements under a finite laser beam spot [2].

High frequency resonator measurements

Supplementary figure S4 shows the experimental set-up for radio-frequency read-out and operation. We use three terminals to operate our device. Source and drain electrode where attached to a suspended graphene layer that covers a centimeter-scale array of openings. Highly doped p-type silicon was used as a global back-gate in order to simultaneously operate each device on the chip. A lock-in approach was employed to measure the distance dependent gating effect on graphene (Supplementary figure S4) following previous reports [3].

Derivation of voltage dependence for PUVD

We identify the transport condition between the graphene layers by investigating the current-voltage behavior. We observe that conduction proceeds by direct tunneling up to 8V before transitioning to Fowler-Nordheim tunneling (Supplementary figure S6 (a)). [4]. This surprisingly high value suggests that a large series resistance exists between the graphene/graphene junction and the electrodes.

We estimate the voltage at the junction by considering the tunneling resistance for graphene/hBN/graphene junctions[5] and the directly measured graphene sheet resistance ($980\Omega/sq$). Under these conditions, 90% of the external voltage drops on the graphene leads and the internal electric field is below 3V/nm throughout the experiments. This field strength is within the range, where ferroelectric polarization becomes stable but below the critical fields that lead to dissociation of water and electric breakdown of the dielectric (Supplementary figure S6(b)).[6] [7].

In the direct tunneling regime, the tunneling current in a ferroelectric junction can be described by: [8]

$$J_{DT} = C \frac{\exp\left(\alpha\left(\left(\phi_2 - \frac{eV}{2}\right)^{\frac{3}{2}} - \left(\phi_1 + \frac{eV}{2}\right)^{\frac{3}{2}}\right)\right)}{\alpha^2\left(\sqrt{\phi_2 - \frac{eV}{2}} - \sqrt{\phi_1 + \frac{eV}{2}}\right)^2} \sinh\left(\frac{3eV}{4}\alpha\left(\sqrt{\phi_2 - \frac{eV}{2}} - \sqrt{\phi_1 + \frac{eV}{2}}\right)\right)$$

In the forward bias regime this formula can be approximated by[9]

$$J = J_T\left(\phi \exp\left(-A\sqrt{\phi}\right) - (\phi + qV)\exp\left(-A\sqrt{(\phi + qV)}\right)\right)$$

Fits to this formula reveal the tunneling prefactor J_T, the tunneling constant A and the barrier height ϕ.

The change in barrier height can be related to the ferroelectric polarization of ice through[10]

$$\Delta\phi = cfp_z$$

where c = 2.44 in the units of volts (V) per debye (D), p_z is the water molecule's dipole moment, and f is concentration of aligned dipoles.

The tunneling prefactor can be furthermore employed to extract the tunneling gap size assuming a unity correction factor[11]

$$J_T = e/2\pi h(\Delta s)^2$$

RF measurements

We employ a vector-network analyzer to extract the complex reflection and transmission coefficients, S11 and S21 for drum-type switch devices (Figure 1(b)). From these measurements we can calculate the dielectric constant following previous reports[12] and the real part of this parameter is shown in Figure 2(e). The frequency dependence of the dielectric constant in a single-oscillator picture is given by the Debye function [13]

$$\varepsilon^*(\omega) = \varepsilon'(\omega) - i\varepsilon''(\omega) = \varepsilon_\infty + \frac{\varepsilon_S - \varepsilon_\infty}{1 + i\omega\tau_0}$$

and we employ its real part to extract the response time τ through fitting to

$$\varepsilon'(\omega) = \varepsilon_\infty + \frac{\varepsilon_S - \varepsilon_\infty}{1 + \omega^2 \tau_0^2}$$

Details on ab-initio simulation

The density functional theory (DFT) calculations were performed using the Vienna ab-initio simulation package (VASP) [14-16]. The Perdew-Burke-Ernzerhof (PBE) exchange-correlation functional[17] was used to perform molecular dynamics and structural optimization. For spot checks of energy ordering of different structures the hybrid PBE0 approximation was also used[18]. The D3 dispersion correction of Grimme[19] using the damping function proposed by Becke and co-workers[20] was employed in all the calculations. The plane-wave basis-set cut-off was set to ~400eV and the dipole of the structure in direction perpendicular to graphene was corrected both in energy and potential[21].

To identify potential ice phases under graphene confinement, we have surveyed a large number of possible geometries in different unit cells, different net dipole orientations (parallel vs perpendicular), and different densities using an automated random structure search at forcefield level. Several low-energy structures for each setting were then selected and reoptimized at the PBE-D3 level. The thus obtained low energy ice phases agree with previous literature reports and are summarized in Suppl. Table 1.

We have tested the stability of the zero-dipole "Cairo" and "hexagonal" phases that Chen et al. identified as the most stable for zero or low pressures[22]. The unit cell for Cairo structure was a=29.652 Å, b=9.783 Å, with 112 carbon atoms in each layer and 36 water molecules in the ice layer. For hexagonal ice of Chen at el., the unit cell had parameters a= 16.944Å, b= 19.565 Å with 128 carbon atoms and 32/ 64 water molecules for single/bilayer ice, respectively. The same unit cell was used for the HD-HMI structure.[23] The structures identified by Chen have no dipole. Initial structures with a dipole were obtained by rotating OH bonds that point to -z to +z direction (see Supplementary figure S7). Subsequent optimization was performed using PBE-D3. The ice XI structure and HD-fRMI[24] used a=16.944 Å, b=14.674 Å, =30 Å.

We also tested a structure with dipoles aligned and perpendicular to graphene and no hydrogen bonds (last line of T1, Fig S7a). This structure is not stable without an external electric field due to the absence of hydrogen bonds between water molecules, which is consistent with results obtained for single water adsorption on graphene[24] Upon optimization, it transforms to the ferroelectric ice XI structure. This “all-dipole-aligned” structure as well as the ice XI structure and HD-fRMI[23] used a=16.944 Å, b=14.674 Å *c=30 Å.*

We furthermore optimize the dipole-oriented structures under different applied electric fields. In the zero-field case, we observe that the modification of the total energy by a formed dipole, either in-plane or out-of-plane, was negligible (see Suppl. Table 1). This observation agrees with previous simulations on the energetic degeneracy of ice Ih and ice XI phases and originates from the small energy change of rotating hydrogen bonds.

Based on these results, the formation of dipole-oriented ice does not occur spontaneously but requires application of an electric field. Our simulations show that ferroelectric ice becomes favorable at fields in excess of 2 V/nm which agrees with simulations by Quian et al.[25]

Once formed, the ferroelectric phase is remarkably stable and an energy barrier of 0.2eV per water molecule was calculated for the reorientation of a dipole from the ferroelectric phase.

The barrier was obtained using the nudged elastic band method [26] with 5 images between the initial and final structures. As with the other calculations, the PBE-D3 functional was used to

obtain the energies. The barrier was obtained in the large simulation cell containing 32 water molecules.

Bilayer ice was built by stacking the obtained net-dipole structure. To form the zero-dipole structure, we again manually rotated the OH bonds to form a structure where no hydrogen interacts with the graphene. All the structures were optimized to find the energy minimum.

Designation	Stability/water molecule (meV)			
	No dipole	Out-of-plane	In-plane	In-plane+out-of-plane
Chen Cairo[22]	-663	-652		
Chen hex[22]	-663	-658	-661	-657
Ice XI	-666			-648
HD-fRMI[23]	-618			
HD-HMI[23]	Transforms into "Chen hex" upon optimization.			
All dipoles aligned		- 193, transforms to the ferroelectric ice XI structure upon optimization		

Table T1 Overview of stability of different ice phases

Details of Molecular Dynamics Simulation

MD simulations were done in VASP on an NVT ensemble using Andersen thermostat with collision probability of 0.1 per atom and time step. Deuterium mass was used for hydrogens and the time step was 1.0 fs with at least 17 ps run times. The distance between layers is approx. 7.2 Å for the single layer ice and 11.4 Å for the two-layer ice. The simulation cell is cubic with a=16.944 Å and b=14.674 Å, c=30 Å to form a vacuum layer. The graphene unit cell size was chosen to be close to the unit cell of ice and contained 96 carbon atoms per layer and 32 water molecules per layer. Dipole corrections (in energy and potential) along z were used. The plane-wave basis-set cut-off was 400 eV.

Estimating the numbers of distinct storage levels

In the proposed multilevel storage scheme, the input signal is represented by the priming voltage which encodes a resistance value that can be read out at low voltages. Thus the number of levels (n) is determined by the precision of the measured conductivity ΔR

$$n = (R_{max} - R_{min})/\Delta R$$

In our case, the precision is limited by the deviation of the measured value from the predicted one. Using a power-dependence to fit the relation between the priming voltage and resistance observed in Figure 3(c) we obtain

$$\Delta R = |R_{meas}(V) - 3 \times 10^9 V^{-4}|$$

The average ΔR in Figure 3(c) was $8.8k\Omega$ and the resistance range for a priming voltage range of 20V was $130k\Omega$ which yields 15 distinct levels of storage. Device optimization could increase this value significantly.

<u>Estimation of stiction force</u>

To calculate the restoring force upon deflection, we employ an analytical formula [27]:

$$F = 2\sigma_0^{2D}\, a \left(\frac{\delta}{a}\right) + \frac{8}{6(1-\nu^2)} E^{2D} a \left(\frac{\delta}{a}\right)^3$$

where σ_0^{2D}, E^{2D} are the 2D pretension and Young's Modulus of the membrane, respectively, ν is the membrane's Poisson ratio, a the hole diameter, δ the deflection. Using reported values for graphene [27] we obtain a force of 4nN for a deflection of 20nm.

This force can be related to the van-der-Waals force [28] at the graphene/graphene interface at ambient conditions and we find that a circular contact with 8nm radius would provide sufficient stiction to maintain contact between the two membranes.

<u>Evaluation of mechanical switching performance</u>

An important parameter to evaluate the power efficiency of transistors and mechanical switches is the on-off ratio between high resistance and low-resistance states [29]. We observe a memristive effect on the on-off ratio which can be tuned through the prior polarization (Supplementary figure S8) and extract a maximum ratio of 10^6.

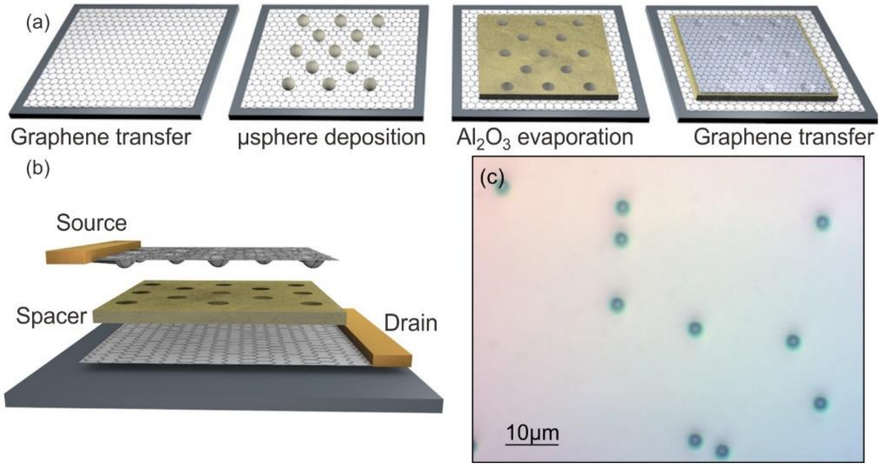

Supplementary figure 1. (a) Schematic of fabrication process, (b) Device schematic, (c) optical micrograph of device after fabrication

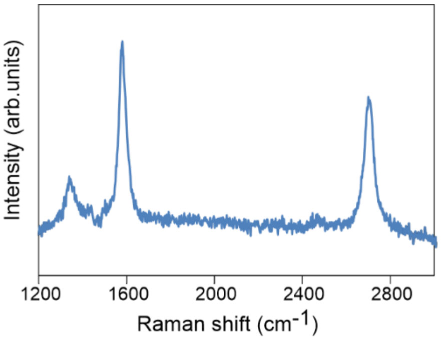

Supplementary figure 2. Representative Raman spectrum of suspended graphene

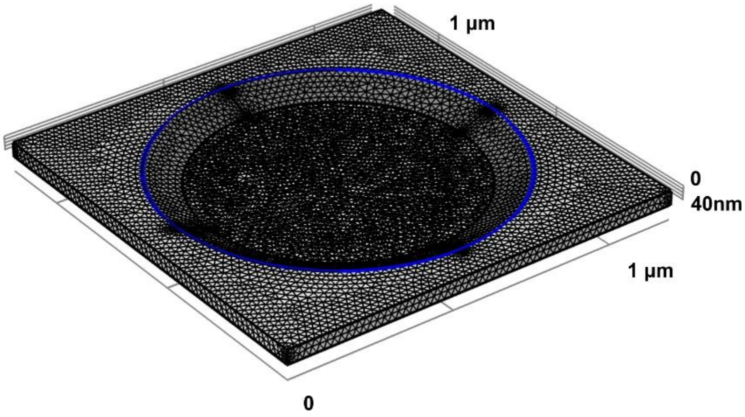

Supplementary figure 3 Finite element simulation and mesh

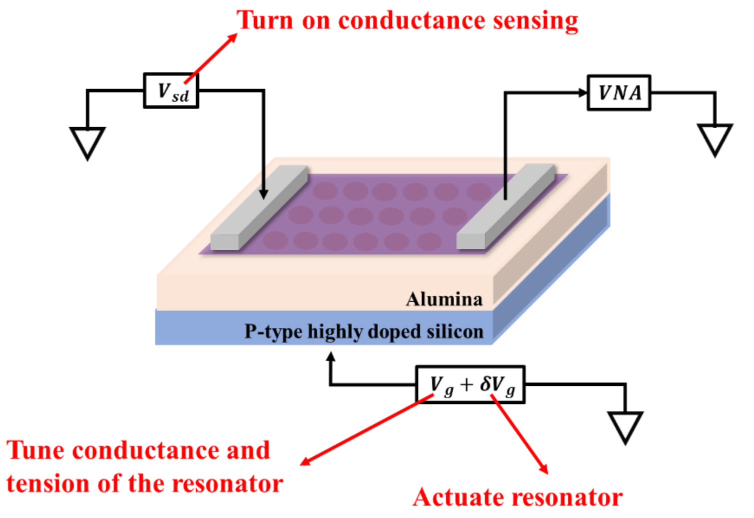

Supplementary figure 4 Schematic of resonator measurement setup

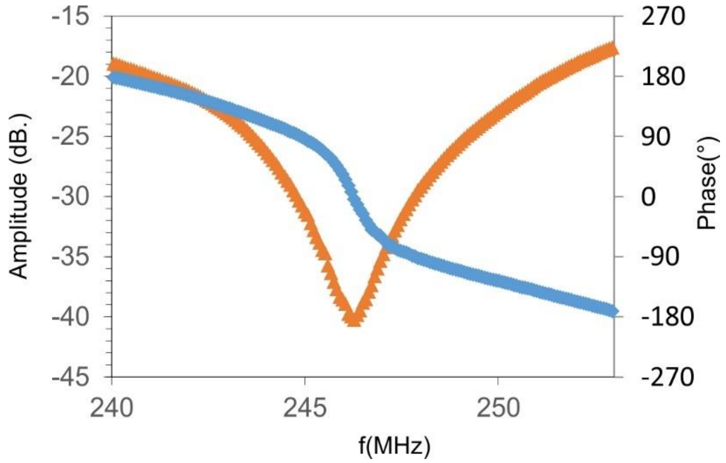

Supplementary figure 5 Frequency dependent measurement of resonators

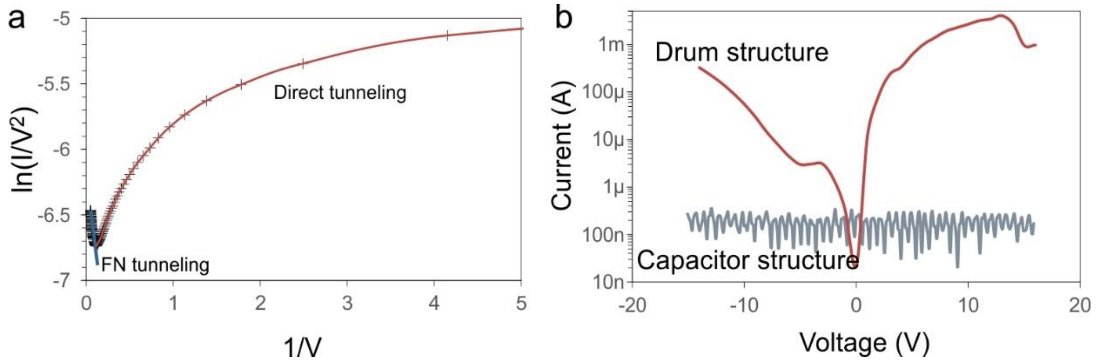

Supplementary figure 6 (a) Fowler-Nordheim plot representation of IV, (b) comparison of here employed drum structure and dummy structure consisting of graphene/dielectric/graphene without openings

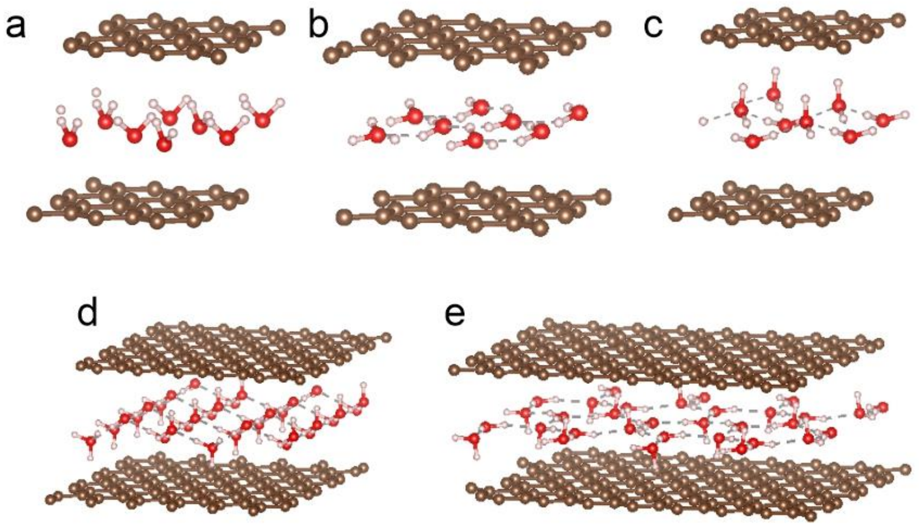

Supplementary figure 7 Examples of investigated ice phases (a) all dipoles aligned (b) HD-fRMI (c) HF-HMI (d) zero-dipole ice XI (e) Chen Cairo

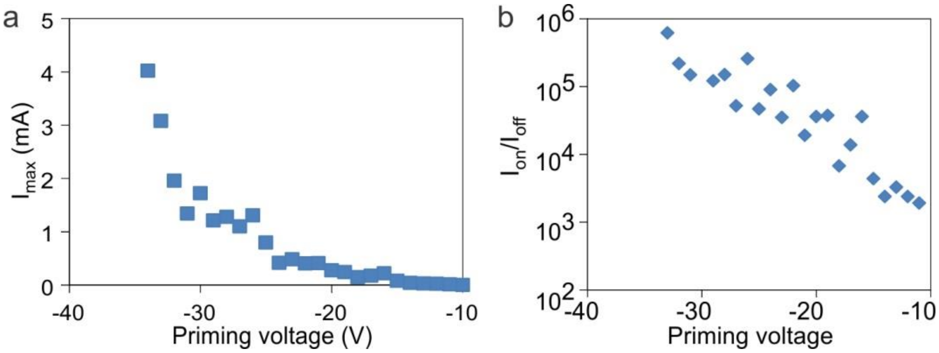

Supplementary figure 7 On/off ratio vs. priming voltage

Supplementary references

1. Hu, Z.; Lu, X., *Mechanical properties of carbon nanotubes and graphene*. Amsterdam: Elsevier: 2014.
2. Hofmann, M.; Hsieh, Y. P.; Liang, C. T.; Chen, Y. F., Size effects on phonon localization and Raman enhancement in silicon nanotips. *J Raman Spectrosc* **2013,** *44* (1), 81-85.
3. Chen, C.; Lee, S.; Deshpande, V. V.; Lee, G. H.; Lekas, M.; Shepard, K.; Hone, J., Graphene mechanical oscillators with tunable frequency. *Nat Nanotechnol* **2013,** *8* (12), 923-7.
4. Beebe, J. M.; Kim, B.; Gadzuk, J. W.; Frisbie, C. D.; Kushmerick, J. G., Transition from direct tunneling to field emission in metal-molecule-metal junctions. *Physical Review Letters* **2006,** *97* (2).
5. Britnell, L.; Gorbachev, R. V.; Jalil, R.; Belle, B. D.; Schedin, F.; Katsnelson, M. I.; Eaves, L.; Morozov, S. V.; Mayorov, A. S.; Peres, N. M. R.; Neto, A. H. C.; Leist, J.; Geim, A. K.; Ponomarenko, L. A.; Novoselov, K. S., Electron Tunneling through Ultrathin Boron Nitride Crystalline Barriers. *Nano Letters* **2012,** *12* (3), 1707-1710.
6. Zhu, W. D.; Huang, Y. Y.; Zhu, C. Q.; Wu, H. H.; Wang, L.; Bai, J.; Yang, J. L.; Francisco, J. S.; Zhao, J. J.; Yuan, L. F.; Zeng, X. C., Room temperature electrofreezing of water yields a missing dense ice phase in the phase diagram. *Nat Commun* **2019,** *10*.
7. Saitta, A. M.; Saija, F.; Giaquinta, P. V., Ab initio molecular dynamics study of dissociation of water under an electric field. *Abstr Pap Am Chem S* **2012,** *244*.
8. Gruverman, A.; Wu, D.; Lu, H.; Wang, Y.; Jang, H. W.; Folkman, C. M.; Zhuravlev, M. Y.; Felker, D.; Rzchowski, M.; Eom, C. B.; Tsymbal, E. Y., Tunneling Electroresistance Effect in Ferroelectric Tunnel Junctions at the Nanoscale. *Nano Letters* **2009,** *9* (10), 3539-3543.
9. Kumar, M.; Georgiadou, D. G.; Seitkhan, A.; Loganathan, K.; Yengel, E.; Faber, H.; Naphade, D.; Basu, A.; Anthopoulos, T. D.; Asadi, K., Colossal Tunneling Electroresistance in Co-Planar Polymer Ferroelectric Tunnel Junctions. *Adv Electron Mater* **2020,** *6* (2).
10. Parkkinen, P.; Riikonen, S.; Halonen, L., Ice XI: Not That Ferroelectric. *J Phys Chem C* **2014,** *118* (45), 26264-26275.
11. Simmons, J. G., Generalized Formula for the Electric Tunnel Effect between Similar Electrodes Separated by a Thin Insulating Film. *J Appl Phys* **1963,** *34* (6), 1793-1803.
12. Lu, Z. J.; Lanagan, M.; Manias, E.; Macdonald, D. D., Two-Port Transmission Line Technique for Dielectric Property Characterization of Polymer Electrolyte Membranes. *J Phys Chem B* **2009,** *113* (41), 13551-13559.
13. Iglesias, T. P.; Vilao, G.; Reis, J. C. R., An approach to the interpretation of Cole-Davidson and Cole-Cole dielectric functions. *J Appl Phys* **2017,** *122* (7).
14. Kresse, G., Ab-Initio Molecular-Dynamics for Liquid-Metals. *J Non-Cryst Solids* **1995,** *193*, 222-229.
15. Kresse, G.; Furthmuller, J., Efficiency of ab-initio total energy calculations for metals and semiconductors using a plane-wave basis set. *Comp Mater Sci* **1996,** *6* (1), 15-50.
16. Kresse, G.; Joubert, D., From ultrasoft pseudopotentials to the projector augmented-wave method. *Physical Review B* **1999,** *59* (3), 1758-1775.
17. Perdew, J. P.; Burke, K.; Ernzerhof, M., Generalized gradient approximation made simple. *Physical Review Letters* **1996,** *77* (18), 3865-3868.
18. Adamo, C.; Barone, V., Toward reliable density functional methods without adjustable parameters: The PBE0 model. *J Chem Phys* **1999,** *110* (13), 6158-6170.

19. Grimme, S.; Antony, J.; Ehrlich, S.; Krieg, H., A consistent and accurate ab initio parametrization of density functional dispersion correction (DFT-D) for the 94 elements H-Pu. *J Chem Phys* **2010,** *132* (15).
20. Becke, A. D.; Johnson, E. R., A density-functional model of the dispersion interaction. *J Chem Phys* **2005,** *123* (15).
21. Paier, J.; Hirschl, R.; Marsman, M.; Kresse, G., The Perdew-Burke-Ernzerhof exchange-correlation functional applied to the G2-1 test set using a plane-wave basis set. *J Chem Phys* **2005,** *122* (23).
22. Chen, J.; Schusteritsch, G.; Pickard, C. J.; Salzmann, C. G.; Michaelides, A., Two Dimensional Ice from First Principles: Structures and Phase Transitions. *Phys Rev Lett* **2016,** *116* (2).
23. Zhao, W. H.; Bai, J.; Yuan, L. F.; Yang, J. L.; Zeng, X. C., Ferroelectric hexagonal and rhombic monolayer ice phases. *Chem Sci* **2014,** *5* (5), 1757-1764.
24. Brandenburg, J. G.; Zen, A.; Fitzner, M.; Ramberger, B.; Kresse, G.; Tsatsoulis, T.; Gruneis, A.; Michaelides, A.; Alfe, D., Physisorption of Water on Graphene: Subchemical Accuracy from Many-Body Electronic Structure Methods. *J Phys Chem Lett* **2019,** *10* (3), 358-368.
25. Qian, Z.; Wei, G., Electric-Field-Induced Phase Transition of Confined Water Nanofilms between Two Graphene Sheets. *The Journal of Physical Chemistry A* **2014,** *118* (39), 8922-8928.
26. Henkelman, G.; Uberuaga, B. P.; Jonsson, H., A climbing image nudged elastic band method for finding saddle points and minimum energy paths. *J Chem Phys* **2000,** *113* (22), 9901-9904.
27. Lee, C.; Wei, X. D.; Kysar, J. W.; Hone, J., Measurement of the elastic properties and intrinsic strength of monolayer graphene. *Science* **2008,** *321* (5887), 385-388.
28. Rokni, H.; Lu, W., Direct measurements of interfacial adhesion in 2D materials and van der Waals heterostructures in ambient air. *Nat Commun* **2020,** *11* (1).
29. Lee, J. H.; Shin, D. H.; Yang, H.; Jeong, N. B.; Park, D. H.; Watanabe, K.; Taniguchi, T.; Kim, E.; Lee, S. W.; Jhang, S. H.; Park, B. H.; Kuk, Y.; Chung, H. J., Semiconductor-less vertical transistor with I-ON/I-OFF of 10(6). *Nat Commun* **2021,** *12* (1).
